# A phase II study of neoadjuvant capecitabine, oxaliplatin, and irinotecan (XELOXIRI) in patients with locally advanced rectal cancer

**DOI:** 10.1002/ags3.12600

**Published:** 2022-07-15

**Authors:** Chu Matsuda, Toshihiro Kudo, Yoshihiro Morimoto, Yoshinori Kagawa, Mitsuyoshi Tei, Yoshihito Ide, Norikatsu Miyoshi, Hidekazu Takahashi, Mamoru Uemura, Ichiro Takemasa, Taroh Satoh, Tsunekazu Mizushima, Kohei Murata, Yuichiro Doki, Hidetoshi Eguchi, Multicenter Clinical Study Group of Osaka, Colorectal Cancer Treatment Group (MSCSGO)

**Affiliations:** ^1^ Department of Gastroenterological Surgery, Graduate School of Medicine Osaka University Osaka Japan; ^2^ Department of Surgery, Osaka International Cancer Institute Osaka Prefectural Hospital Organization Osaka Japan; ^3^ Department of Medical Oncology, Osaka International Cancer Institute Osaka Prefectural Hospital Organization Osaka Japan; ^4^ Department of Gastroenterological Surgery Kansai Rosai Hospital Amagasaki Japan; ^5^ Department of Gastroenterological Surgery Osaka General Medical Center Osaka Japan; ^6^ Department of Surgery Osaka Rosai Hospital Osaka Japan; ^7^ Department of Surgery Yao Municipal Hospital Osaka Japan; ^8^ Department of Surgery Japan Community Health care Organization (JCHO) Osaka Hospital Osaka Japan; ^9^ Department of Surgery, Surgical Oncology and Science Sapporo Medical University Sapporo Japan; ^10^ Department of Therapeutics for Inflammatory Bowel Diseases, Graduate School of Medicine Osaka University Osaka Japan

**Keywords:** capecitabine, irinotecan, neoadjuvant chemotherapy, oxaliplatin, rectal cancer

## Abstract

**Purpose:**

Addition of perioperative multi‐agent chemotherapy to the treatment strategy for locally advanced rectal cancer (LARC) may be a promising option. We conducted a phase II study to evaluate the safety and efficacy of capecitabine combined with oxaliplatin and irinotecan (XELOXIRI) as triplet neoadjuvant chemotherapy in patients with LARC.

**Methods:**

Patients received neoadjuvant irinotecan and oxaliplatin and capecitabine and then underwent total mesorectal excision. The primary study endpoint was the pathological complete response (pCR) rate.

**Results:**

Between June 2013 and December 2016, 55 patients were enrolled in the study. Forty‐two (77.8%) of 54 completed the study protocol. The pCR rate was 7.7% (95% CI 3.0% to 18.2%). The 3‐year local recurrence rate was 3.9%, the 3‐year disease‐free survival (DFS) rate was 77.3, and the 3‐year overall survival rate was 96.0%.

**Conclusion:**

XELOXIRI neoadjuvant chemotherapy appears to be feasible and efficacious for patients with LARC. Although neoadjuvant XELOXIRI alone did not yield our expected pCR rate, the local recurrence rate, 3‐year DFS, and measures of safety met current standards.

## Graphical abstract

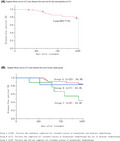

## 1 INTRODUCTION

In cases of locally advanced rectal cancer (LARC), local recurrence and/or distant metastasis often occur despite R0 resection. Thus, outcomes are unsatisfactory when treatment is limited to total mesorectal excision (TME), which is a standard surgical procedure for rectal cancer.^1^ For this reason, LARC is usually treated not only by surgery but also perioperatively by combined radiation and chemotherapy.

In Western countries, the widely accepted standard treatment for LARC consists of neoadjuvant long‐course chemoradiation or short‐course hypofractionated radiotherapy followed by TME and adjuvant chemotherapy. In Japan, the standard treatment for rectal cancer remains upfront surgery with lateral pelvic lymph node dissection followed by adjuvant chemotherapy. Although neoadjuvant chemoradiotherapy (NCRT) has been shown to significantly decrease the locoregional recurrence rate in cases of LARC, significant effects on distant recurrence and overall survival (OS) rates have not been found.^2^, ^3^ In addition, NCRT is associated with long‐term bladder and sexual dysfunction, fecal incontinence, and myelosuppression.^4^ Application of multi‐agent systemic chemotherapy as neoadjuvant chemotherapy (NAC) may allow patients to escape the early and late complications of radiation therapy as well as the development of distant metastasis and locoregional recurrence.

Several studies have been conducted to evaluate the efficacy and safety of NAC alone for LARC,^5^, ^6^, ^7^ and clinical trials of NAC (CAPOX plus bevacizumab [BEV], perioperative CAPOX), aimed at improving disease‐free survival (DFS) and OS of patients with rectal cancer, have been conducted.^8^, ^9^ However, results of these trials were unsatisfactory.

Meanwhile, a triplet combination of fluoropyrimidine, oxaliplatin, and irinotecan, either the FOFOXIRI or XELOXIRI regimen, administered to patients with metastatic colorectal cancer was shown to yield a higher response rate than that of doublet chemotherapy.^10^, ^11^ Few studies have investigated efficacy of a triplet regimen administered as NAC in patients with LARC. Considering triplet combinations as more powerful than other regimens, a clinical trial of the triplet combination XELOXIRI was initiated, and members of our study group have reported results of phase I of the trial.^12^ Herein, we report the results of phase II, conducted to confirm the safety of XELOXIRI administered as NAC in patients with LARC and to evaluate the efficacy of XELOXIRI administered as NAC.

## 2 PATIENTS AND METHODS

### 2.1 Patients and eligibility criteria

Eligibility criteria were as follows: (1) age over 20 years; (2) Eastern Cooperative Oncology Group (ECOG) performance status of 0 or 1; (3) histologically proven adenocarcinoma of the rectum; (4) resectable clinical stage II (T3 or T4 with N0) or III (any T and N1‐3), with a positive node defined as ≥0.7 mm in a short‐axis diameter on imaging and the primary tumor located either above or below the peritoneal reflection and the inferior tumor margin located within 12 cm of the anal verge; and (5) adequate organ function defined by a white blood cell count ≥4000 × 10^9^/L, hemoglobin ≥9.0 g/dL, platelet count ≥100 × 10^9^/L, total bilirubin ≤1.5 mg/dL, serum transaminases ≤150 IU/L, and serum creatinine ≤1.5 mg/dL.

Exclusion criteria were as follows: (1) major surgery, radiation therapy, and prior chemotherapy within 4 weeks of inclusion in the trial; (2) a history of interstitial pneumonia, watery diarrhea, evidence of active infection (including positivity for hepatitis B surface antigen), or presence of a severe, systemic disease (e.g. heart failure, hepatic failure, ulcer, uncontrolled diabetes mellitus); (4) presence of another malignancy within the previous 5 years; (5) pregnancy or breast feeding; (6) a homozygous UGT1A1*6/*6 or UGT1A1*28/*28 genotype, or heterozygous UGT1A1*6/*28 genotype; and (7) peripheral neuropathy grade ≥2 according to National Cancer Institute‐Common Toxicity Criteria (NCI‐CTC) v. 4.0.

Four institutions participated in this study. The ethics committee at each of the participating institutions approved this phase II study, and all patients provided written informed consent before any specific study procedure was undertaken.

### 2.2 Study design and treatment

Patients enrolled in the study described having received intravenous oxaliplatin (85 mg/m^2^) and intravenous irinotecan (150 mg/m^2^) on day 1 and oral capecitabine (1000 mg/m^2^) twice daily on days 1‐7 followed by 7 days of rest, according to the recommended dose established in the previously reported phase I study.^12^ Irinotecan in 5% dextrose (250 ml) was administered over a 90 min period and followed immediately by concomitant infusion of oxaliplatin in 5% dextrose (250 ml) and 200 mg/m^2^ leucovorin in 5% dextrose (250 ml) over a 2 h period through a Y connector. Treatment was repeated every 2 weeks for six cycles or until disease progression. Surgery, either TME or tumor‐specific mesorectal excision, was performed 4‐8 weeks after chemotherapy ended. Lateral lymph node dissection was performed at the surgeon's discretion. In addition to the treatment protocol (NAC plus surgery), patients received adjuvant chemotherapy (referred to herein as CAPOX), initiated within 8 weeks after surgery, consisting of oral capecitabine (1000 mg/m^2^) twice daily on days 1 through 14 and intravenous oxaliplatin (130 mg/m^2^) on day 1 every 3 weeks for four cycles.

### 2.3 Dose adjustments

Adverse events were assessed according to the Common Toxicity Criteria of the National Cancer Institute (version 4.0). If a patient developed a grade 4 hematological and/or grade 3 or higher non‐hematological adverse event, the dose capecitabine and/or oxaliplatin and/or irinotecan was reduced by 25% for all subsequent cycles. In patients who developed allergic reactions or laryngeal spasm syndrome, duration of the oxaliplatin infusion was increased to 4‐6 h. Treatment was delayed for up to 3 weeks if a patient's absolute neutrophil count was lower than 1500/μl, or their platelet count was lower than 75,000/μl. Patients who required more than 3 weeks recovery from an adverse reaction were excluded from the study.

### 2.4 Pathological analysis

Standard pathological analysis was performed on all resected specimens at each of the participating institutions. Tumor regression was graded on the basis of a previously described classification system.^13^ This was done to determine the effect of NAC, with effects graded as follows: grade 0, no effect; grade 1a, tumor cell necrosis, or degeneration in less than 1/3 of the entire lesion; grade 1b, tumor cell necrosis, degeneration and lytic change in more than 1/3 but less than 2/3 of the entire lesion; grade 2, prominent tumor cell necrosis, degeneration, lytic change and/or disappearance of tumor cells in more than 2/3 of the entire lesion but presence of some viable tumor cells; and grade 3, absence of viable tumor cells.

### 2.5 Endpoints and statistical analysis

The primary study endpoint was the pathological complete response (pCR) rate. Secondary endpoints were 3‐year DFS, 3‐year OS, 3‐year local recurrence rate, the R0 resection rate, and safety in terms of adverse events.

The pCR rate has been reported to range from 10% to 20% among patients who have undergone NCRT.^14^, ^15^, ^16^, ^17^ We based the sample size of the study described herein on an expected pCR rate of 18% and a threshold pCR rate of 5% to detect differences at a two‐sided alpha error of 0.05 and a statistical power of 0.2. As such, the planned sample size was 48 patients, allowing for a 10% dropout rate.

DFS was calculated from the start of treatment to disease progression or to death from any cause, and OS was calculated from the start of treatment to death from any cause, using the Kaplan‐Meier method. DFS and OS were compared between subgroups, and differences were analyzed by log‐rank test. All statistical analyses were performed with JMP 13 (SAS Institute,).

## 3 RESULTS

### 3.1 Patient characteristics

**FIGURE 1:**
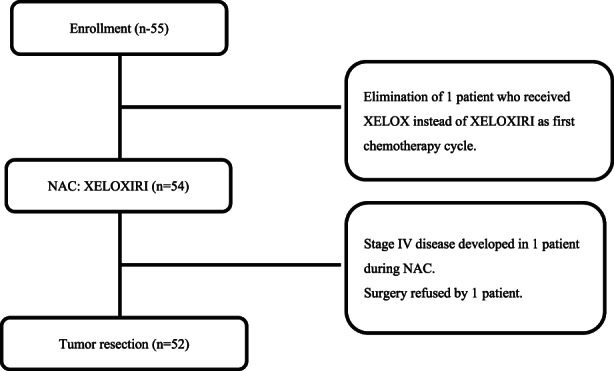
Flow diagram of patients' progress through the study

Fifty‐five patients with LARC were enrolled in the study from the four institutions between June 2013 and December 2016. A flow diagram showing patient enrollment and their progression through the study protocol is given in Figure 1.

Clinical characteristics of the 55 enrolled patients are summarized in Table 1. Median age was 63.5 years (range, 32‐77 years), and 44 (80.0%) were men. All but one patient had an ECOG performance status of 0. Pretreatment carcinoembryonic antigen concentrations ranged from 0.6 to 153.4 (median 4.0) ng/ml. Median distance from the tumor to the anal verge was 5.0 (range, 0‐10.0) cm. Median tumor diameter was 4.0 (range, 2.0‐10.0) cm. The depth of the tumor was cT2 in one patient, cT3 in 69.1% of the patients, cT4a in 21.8%, and cT4b in four patients. Tumors of T4b invaded the prostate in two patients, the seminal vesicles in one patient, and the bladder in one patient, according to MRI. The disease was of cStage II in 31 patients (56.4%) and of cStage III in 24 (43.6%).

**TABLE 1:** Patients' clinical characteristics (*n* = 55)

Age, *years*	63.5 (32–77)
Sex, *M/F*	44/11
ECOG performance status, *0/1*	54/1
CEA, *ng/mL*	4.0 (0.6–153.4)
Distance from tumor to anal verge, *cm*	5.0 (0–10.0)
Tumor diameter, *cm*	4.0 (2.0–10.0)
Location of primary tumor, *Ra/Rb*	10/45
Clinical T stage, *T2/T3/T4a/T4b*	1/38/12/4
Clinical N stage, *N0/N1/N2/N3/NX*	30/13/5/5/2
Clinical stage, *II/IIIA/IIIB*	31/14/10
Histology, *tub1 or 2/por or muc or sig*	52/3
Comorbidity, *+/−*	22/33
Hypertension, *+/−*	17/38
Diabetes mellitus, *+/−*	5/50
Cerebrovascular disease, *+/−*	0/55
Ischemic heart disease, *+/−*	1/54
Neuropathy, *+/−*	0/55
Proteinuria, *+/−*	0/55
Other, *+/−*	6/49

*Note*: Data are shown as median (range) values or numbers of patients.

Abbreviations: *CEA*, carcinoembryonic antigen; *ECOG*, Eastern Cooperative Oncology Group; *Ra/Rb* above/below the peritoneal reflection.

### 3.2 Treatment

Fifty‐four of the 55 patients underwent NAC as intended. One patient received XELOX in the first cycle instead of XELOXIRI. Forty‐five (83.3%) of the 54 patients who underwent NAC received the full six courses. As noted above, one patient was found during NAC to have stage IV disease (paraaortic lymph nodes and left subclavian lymph nodes metastasis). Another patient declined to undergo the surgery. Thus, 52 patients underwent tumor resection, and 44 of these 52 patients (84.6%) underwent adjuvant chemotherapy, with 38 (38/44, 86.4%) having completed the full four courses of CAPOX (Table 2).

**TABLE 2:** Treatment variables and outcomes

Neoadjuvant chemotherapy (*n* = 54)	
*6/5/4/3/2/1 cycles*	45/0/1/5/1/2
Surgical outcomes (*n* = 52)	
Operative procedure	
Low anterior resection	25
Intersphincteric resection	19
Abdominoperineal resection	8
Combined resection	0
Surgical approach, *laparoscopic/open*	47/5
Lateral lymph node dissection (*n* = 35), *bilateral/unilateral/none*	29/6/17
Operation time, min	414 (201‐917)
Blood loss volume, *mL*	100 (0‐2310)
Residual tumor status, *R0/R1/R2*	49/2/1
Lateral lymph node metastasis, *+/−*	7/28
Postoperative complication	
Any complicatios	17
Major complications *	5
Ileus	5 (3*)
Urinary disfunction	7 (1*)
Anastomotic leakage	1 (1*)
Wound infection	2 (0*)
Nerve injury (Obturator nerve, Sciatic nerve)	2 (0*)
Mortality	0
Pathological outcomes (*n* = 52)	
pCR	4
Histologic response, *Grade 0/1a/1b/2/3*	1/15/18/14/4
ypStage, *0/I/II/III/IV*	4/14/15/18/1
Adjuvant chemotherapy, *4/3/2/1/ cycles*	38/3/1/2/8

*Note*: Number of patients or median (range) values are shown.

*pCR,* pathological complete response.

*Grade 3 according to the Clavien‐Dindo classification.

### 3.3 Perioperative outcomes

#### 3.3.1 Surgical outcomes and R0 resection rate

Perioperative outcomes, including the numbers of NAC cycles undertaken, the numbers and types of surgery performed, results of pathological examination, and the numbers of adjuvant chemotherapy cycles undertaken are shown in Table 2. Laparoscopic surgery was performed in 47 (90.4%) patients, and lateral lymph node dissection was performed in 35 (67.3%) patients. Forty‐nine (94.2%) patients underwent R0 resection. Lateral lymph nodes were positive in seven of the 35 patients in whom lateral lymph node dissection was performed. There were no patients who needed combined resection of adjacent organs.

Seventeen of the 52 patients who underwent surgery (32.7%) experienced any complications within 30 days of surgery. The complications experienced by these patients included ileus (five events, 9.6%), urinary disfunction (seven events, 13.5%), anastomotic leakage (one event, 1.9%), wound infection (two events, 3.8%) and nerve injury (obturator nerve, one event, 1.9%; sciatic nerve, one event, 1.9%). Five patients (9.6%) had major complications of grade 3 according to Clavien‐Dindo classification. No patient had grade 4 or higher complications.

### 3.4 Pathological outcomes

#### 3.4.1 pCR rate and pathological effects

pCR was achieved in four patients (4/52) (7.7% [95% CI 3.0%‐18.2%]), and the final classification was ypStage 0 in four patients, ypStage I in 14 patients, ypStage II in 15 patients, ypStage IIIa in nine patients, ypStage IIIb in nine patients, and ypStage IV in one patient. Pathological stages are shown in relation to patients' baseline T and N stages for the 52 patients who underwent resection in Table 3. The down‐staging rate in terms of T stage was 63.5% (33/52). Eighty percent (12/15) of cT4 tumors were down‐staged after NAC. The down‐staging rate in terms of N stage was 65.4% (34/52).

**TABLE 3:** Final pathological stages in relation to baseline T and N stages (*n* = 52)

	Final pathological stage						
	ypT0	ypTis	ypT1	ypT2	ypT3	ypT4a	Total
Baseline T stage							
T2	0	0	0	0	0	0	0
T3	2	0	0	19	16	0	37
T4a	2	1	1	1	4	3	12
T4b	0	0	0	1	2	0	3
Total	4	1	1	21	22	3	52
	ypN0	ypN1	ypN2	ypN3			
Baseline N stage							
N0	22	3	3	1			29
N1	7	3	1	1			12
N2	1	3	1	0			5
N3	1	0	0	3			4
NX	2	0	0	0			2
Total	33	9	5	5			52

### 3.5 Safety (adverse events)

Adverse events are shown in relation to NAC and to adjuvant chemotherapy in Table 4. The most common NAC‐related adverse events were anemia (59.3%), neutropenia (44.4%), diarrhea (42.6%), and peripheral neuropathy (40.7%). NAC‐related grade ¾ adverse event rates, from highest to lowest, were as follows: neutropenia (25.9%), anorexia (13.0%), diarrhea (11.1%), and anemia (7.4%). No patient suffered grade 3/4 NAC‐related peripheral neuropathy or hand‐foot syndrome. Adjuvant chemotherapy‐related grade 3/4 adverse events occurred in nine patients (20.5%), with grade 3 peripheral neuropathy occurring in four of the nine. One patient died while undergoing adjuvant chemotherapy, with the death attributed to cerebral infarction.

**TABLE 4:** NAC‐ and adjuvant chemotherapy‐related adverse events

	NAC (*n* = 54)		Adjuvant chemotherapy (*n* = 44)		
	Any grade event	Grade 3/4 event	Any grade even	Grade 3/4 event	Grade 5 event
Thrombocytopenia	13 (24.1)	1 (1.9)	10 (22.7)	‐	‐
Anemia	32 (59.3)	4 (7.4)	19 (43.2)	‐	‐
Neutropenia	24 (44.4)	14 (25.9)	8 (18.2)	2 (4.5)	‐
Peripheral neuropathy	22 (40.7)	‐	25 (56.8)	4 (9.1)	‐
Diarrhea	23 (42.6)	6 (11.1)	4 (9.1)	‐	‐
Hand‐foot syndrome	2 (3.7)	‐	1 (2.3)	‐	‐
Anorexia	13 (24.1)	7 (13.0)	5 (11.4)	2 (4.5)	‐
Nausea	3 (5.6)	1 (1.9)	1 (2.3)	‐	‐
Fatigue	6 (11.1)	2 (3.7)	3 (6.8)	‐	‐
Vomiting	1 (1.9)	‐	1 (2.3)	‐	‐
Oral mucositis	2 (3.7)	‐		‐	‐
ALT/AST elevation	‐	1 (1.9)	1 (2.3)	‐	‐
Pulmonary thromboembolism	1 (1.9)	1 (1.9)	‐	‐	‐
Small bowel obstruction	1 (1.9)	‐	‐	‐	‐
Cerebral infarction	‐	1 (1.9)	‐	‐	1 (2.3)
Edema	1 (1.9)		‐	‐	‐
Perianal abscess	‐	1 (1.9)	‐	‐	‐
Dermatitis	1 (1.9)	‐	‐	‐	‐
Dehydration	‐	‐	‐	1 (2.3)	‐

*Note*: Number (and percentage) of patients are shown.

Abbreviations: *ALT*, alanine transaminase; *AST*, aspartate transaminase; *NAC*, neoadjuvant chemotherapy.

### 3.6 Three‐year local recurrence rate

The follow‐up period ranged from 0.45 to 6.0 years (median, 3.45 years). R0 or R1 resection was achieved in 51 patients. Eleven of the 51 patients suffered either metastatic or local recurrence: lung metastasis (*n* = 5), liver metastasis (*n* = 1), ovarian metastasis (*n* = 1), adrenal gland metastasis (*n* = 1), paraaortic lymph node metastasis (*n* = 1) lateral lymph node metastasis (*n* = 1), and local recurrence (*n* = 1). Lateral lymph node dissection had not been performed in the patient with lateral lymph node recurrence. The local recurrence rate was 3.9% (2/51).

### 3.7 Three‐year DFS and 3‐year OS

**FIGURE 2:**
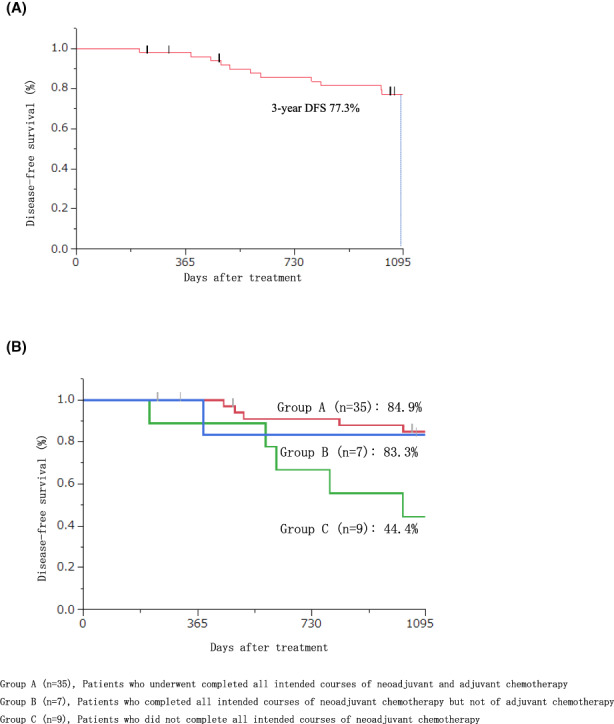
(A) Kaplan‐Meier curves of 3‐year disease‐free survival (DFS) for the total patients (*n* = 51). (B) Kaplan‐Meier curves of 3‐year disease‐free survival (DFS) per study group. Group A (*n* = 35): Patients who completed all intended cycles of neoadjuvant and adjuvant chemotherapy. Group B (*n* = 7): Patients who completed all intended cycles of neoadjuvant chemotherapy but not all courses of adjuvant chemotherapy. Group C (*n* = 9): Patients who did not complete all intended cycles of neoadjuvant chemotherapy

Five of the 54 patients who underwent NAC died, three from cancer, one from cerebral infarction (as noted above), and one from myocardial infarction. Kaplan‐Meier curves for DFS are shown in Figure 2. Three‐year DFS was 77.3%. Three‐year OS was 96.0% (data not shown). We compared DFS between the 35 patients who underwent both NAC and adjuvant chemotherapy (Group A), the seven patients who completed NAC but did not complete adjuvant chemotherapy (Group B) and the nine patients who did not complete NAC (Group C). Three‐year DFS was 84.9%, 83.3%, and 44.4%, respectively. Three‐year DFS of Groups A and B combined, i.e. patients who completed NAC, was 84.7%.

## 4 DISCUSSION

Total neoadjuvant therapy, which includes CRT plus induction or consolidation chemotherapy, is being performed worldwide, and good outcomes have been reported.^18^, ^19^, ^20^, ^21^ The developed multi‐agent chemotherapy is now being used for neoadjuvant chemotherapy with the goal of improving patient prognosis not only by shrinking the local tumor but also by controlling distant metastasis.

A phase III FOWARC randomized controlled study conducted in China to compare CRT and NAC alone for LARC is the first to report long‐term outcomes.^22^ No significant between‐group differences were reported in 3‐year DFS, 3‐year OS, or the 3‐year local recurrence rate (mFOLFOX arm, mFOLFOX with radiation arm, conventional fluorouracil with radiation arm). Although radiation significantly improved the pCR rate (6.6%, 27.5%, and 14.0% in the three arms, respectively), radiation did not improve long‐term outcomes and worsened postoperative anal function. Therefore, NAC alone, by which the toxicities of radiation are eliminated, has been reviewed. Several groups have evaluated efficacy and safety of NAC alone for LARC.^6^, ^7^, ^8^ Schrag et al reported results of NAC alone in 32 patients with intermediate‐risk rectal cancer.^6^ They evaluated the efficacy of neoadjuvant FOLFOX + BEV without radiation followed by TME and reported excellent short‐term outcomes. The pCR rate was 25%. The 4‐year local recurrence rate was 0%, and 4‐year DFS was 84%. We expect the ongoing randomized phase III PROSPECT trial (NAC alone vs chemotherapy plus radiation therapy) to provide additional insight into the usefulness of NAC alone for LARC.^23^

As noted above, the response rate reported for the triplet combination of fluoropyrimidine, oxaliplatin, and irinotecan was higher than that for doublet chemotherapy in patients with metastatic colorectal cancer.^10^, ^11^ Few studies have investigated the activity of any triplet regimen as NAC in patients with LARC.

The BACCHUS trial^24^ is the only randomized clinical trial in which a triplet‐chemotherapy plus BEV in patients with rectal cancer has been investigated.^24^ The trial, comparing neoadjuvant FOLFOX + BEV with FOLFXIRI + BEV for LARC, was conducted in the UK, but unfortunately it was terminated early because of poor accrual. Neoadjuvant FOLFOXIRI + BEV therapy achieved a promising pCR rate (20%) and the therapy was well‐tolerated.

Preoperative use of molecularly targeted drugs is expected to shrink tumors and improve outcomes. However, use of such drugs may increase occurrence of postoperative complications such as anastomotic leakage.^7^ Therefore, the study we describe herein was conducted as a phase II study investigating the efficacy of a triplet NAC regimen that does not include a molecular‐targeted drug.

Our study was of XELOXIRI used for NAC in patients with LARC. The protocol completion rate (i.e. a full course of XELOXIRI + surgery) was 77.8% (42/54). Twenty‐eight (66.7%) of the 42 patients completed a full course of CAPOX as adjuvant chemotherapy. Tumor shrinkage due to NAC may increase the chance of R0 resection. Of our 52 patients who underwent surgery after NAC, R0 resection was achieved in 49 (94.2%). Other NAC trials have yielded an R0 resection rate of 90%‐100%.^7^, ^8^, ^9^, ^10^

Our primary study endpoint was the pCR rate. Pathological CR was observed in four of our 52 patients for a pCR rate of 7.7% (95% CI 3.0%‐18.2%). Reported doublet NAC trials have yielded pCR rates of 2.4%‐13%,^7^, ^8^, ^9^, ^10^ and in the BACCHUS trial, neoadjuvant FOLFOXIRI + BEV therapy yielded a pCR rate of 20%. pCR rates reported from CRT trials have ranged between 10%‐20%.^15^, ^16^, ^17^, ^18^ Although we expected enhanced therapeutic power of a triplet regimen as a NAC without RT, our pCR rate was not as high as expected. One reason for the pCR rate not achieving the expected 18% even though 83.3% of patients received full‐dose preoperative treatment is that we did not use molecular‐targeted drugs to prevent side effects. As another reason, NAC has not been able to achieve the pCR rates attained by treatments including RT, even when the NAC incorporates three drugs. The failure to increase the pCR rate despite the completion of NAC may indicate that preoperative cytotoxic chemotherapeutic agents is limited in terms of what can be achieved. However, tumor regression was good (grade 2 or 3) in 18 patients (34.6%), presumably due to the effect of the triplet chemotherapy.

Although a triplet regimen such as FOLFOXIRI and XELOXIRI is a common regimen for metastatic or recurrent colorectal cancer and its safety and tolerability have been shown,^11^, ^12^ these have not yet been established in the neoadjuvant setting. This triplet regimen has a particularly high rate of grade 3 or higher neutropenia and diarrhea. In this XELOXIRI study, the rate of neutropenia (≥ grade 3) is 25.9%, which is higher than that of the XELOX regimen of 12.5%‐17%.^7^, ^8^ However, it is lower than that of the FOLFOXIRI regimen (50%) and the FOLFIRI regimen (28%) for metastatic or recurrent colorectal cancer.^10^ The rate of diarrhea (≥ grade 3) is 11.1% in this study, which is also higher than that of the XELOX regimen of 2.4%‐3.1%,^7^, ^8^ although it is lower than the FOLFOXIRI regimen (17%‐30%) and the doublet regimen (11%‐14%).^11^, ^12^ In addition, in our study, hematologic toxicity (≥ grade 3) was higher compared with the CRT trials (3.7%‐6%),^25^, ^26^ but there are no toxicities of radiation such as dermatitis, anal pain, and fecal incontinence. Postoperative complications were also less common than with other treatments.^7^, ^8^, ^9^, ^10^, ^11^, ^12^, ^25^, ^26^ Careful dose control is required for neutropenia during XELOXIRI, but generally it is acceptable.

Among our study patients, the 3‐year local recurrence rate was 3.9%. Doublet NAC trials have resulted in local recurrence rates of 7%‐10%,^7^, ^8^, ^9^, ^10^ and CRT trials have resulted in local recurrence rates of 4%‐7.6%.^15^, ^16^, ^17^, ^18^ By comparison, the local recurrence rate among our study patients was extremely low. Local control achieved by triplet XELOXIRI NAC may be comparable to that achieved by CRT. T‐factor down‐staging was detected in 63.5% (33/52) of the patients. Two of the three patients who were diagnosed as T4b before NAC converted to yT3 after resection and the other patient converted to yT2. T‐factor down‐staging may also contribute to the suppression of local recurrence.

Among our total study patients, the 3‐year DFS was 77.3% and the 3‐year OS was 96.0%. The subgroup analysis showed the 3‐year DFS of 42 patients (82.4%) who completed the study protocol was 84.7%. Doublet NAC trials have resulted in 3‐year DFS rates of 71%‐73%,^9^, ^10^ and CRT trials have resulted in 3‐year DFS rates of 71%‐75%.^26^ The FOWARC study mentioned above yielded 3‐year DFS rates of 73.5%, 72.9%, and 77.2% for patients in the NAC arm (mFOLFOX), CRT arm (fluorouracil with RT), and mFOLFOX with the RT arm, respectively, and 3‐year OS rates of 90.7%, 91.3%, and 89.1%, respectively. Our 3‐year DFS and 3‐year OS rates were higher than those of the FOWARC study (Table S1).^22^ The difference might be due to the effectiveness of triplet XELOXIRI, the high adjuvant chemotherapy completion rate, and the high prevalence of lateral lymph node dissection.

In recent years, the development of total neoadjuvant therapy including doublet chemotherapy has progressed, and large‐scale randomized trials have shown improvements in pCR and DFS but not in OS.^27^, ^28^, ^29^ Data from our study indicate that clinical trials of total neoadjuvant therapy, including triplet XELOXIRI, are warranted.

### 4.1 Study limitations

Our data should be interpreted in light of our study limitations. The study was conducted as a single‐arm trial, and the patient group was fairly small. Because of the limited sample size, we were unable to investigate which type of LARC is most susceptible to XELOXIRI used as NAC. Notably, a large phase II/III study (PROSPECT trial) that compares CRT with neoadjuvant FOLFOX chemotherapy is being conducted in the United States. We anticipate that additional trials will be conducted to confirm the efficacy of triplet NAC protocols and any related adverse events.

## 5 CONCLUSION

Although our study did not show improvement in pCR with XELOXIRI used as NAC for LARC, the local recurrence rate, 3‐year DFS, and safety were acceptable. The study was conducted as a prospective single‐arm clinical trial that included a fairly small group of patients. Thus, there remains a need for a larger trial to investigate the effectiveness of triplet NAC for LARC.

## AUTHOR CONTRIBUTIONS

Authors making substantial contributions to conception and design, and/or acquisition of data, and/or analysis and interpretation of data: CM, TK, YM, YK, MT, YI, IT, TS, TM; authors participating in drafting the article or revising it critically for important intellectual content: CM, TK, YM, YK, MT, YI, NM, HT, MU, TM, YD, HE; authors giving final approval of the version to be published: CM, TK, YM, YK, MT, YI, NM, HT, MU, IT, TS, TM, KM, YD, HE.

## CONFLICTS OF INTEREST

I.T., T.M., and Y.D. are Editorial Board Members of AGS.

## ETHICAL APPROVAL

This study was conducted in accordance with the Declaration of Helsinki and Ethical Guidelines for Clinical Studies in Japan, and the protocol was approved by the institutional review boards of all participating medical institutions. This study was registered in the UMIN clinical trials registry. (UMIN‐CTR number UMIN000009974)

## INFORMED CONSENT

The informed consent form stated that the results would be published after completion of the study. Consent of the family is not required at the time of article publication according to Japanese ethical guidelines.

## Supporting information


Table S1
