# Supplementary material for: A phase II study of neoadjuvant capecitabine, oxaliplatin, and irinotecan (XELOXIRI) in patients with locally advanced rectal cancer
Source: Ann Gastroenterol Surg. 2022 Jul 15;7(1):81–90. doi: 10.1002/ags3.12600 (PMC9831889; doi:10.1002/ags3.12600)
Supplement: Supplementary file 1 — Table S1 [file AGS3-7-81-s001.docx]

**Supplementary Table S1**

Comparison of oncological outcomes between FOWARC study and this study

|  | FOWARC Trial * | | | This study |
| --- | --- | --- | --- | --- |
|  | CRT | CRT | NAC | NAC |
|  | FOLFOX6+RT (n=39) | FU+RT (n=46) | FOLFOX6 (n=46) | **XELOXIRI (n=54)** |
| pCR rate (%) | 27.5 | 14 | 6.6 | **7.7** |
| 3-year DFS (%) | 77.2 | 72.9 | 73.5 | **77.3** |
| 3-year Local Rec. rate (%) | 7 | 8 | 8.3 | **3.9** |
| 3-year OS (%) | 89.1 | 91.3 | 90.7 | **96** |

* Deng Y, Chi P, Lan P et al (2016) J Clin Oncol 34(27):3300–3307.
